# Complosome as a new intracellular regulatory network in both normal and malignant hematopoiesis

**DOI:** 10.1038/s41375-025-02613-7

**Published:** 2025-04-23

**Authors:** Mariusz Z. Ratajczak, Adrian Konopko, Justyna Jarczak, Michalina Kazek, Janina Ratajczak, Magdalena Kucia

**Affiliations:** 1 Department of Regenerative Medicine Warsaw Medical University, Warsaw, Poland; 2 Stem Cell Institute at Graham Brown Cancer Center, University of Louisville, Louisville, KY, USA

**Keywords:** Stem-cell research, Haematopoiesis

## Abstract

Hematopoietic cells and lymphocytes arise from a common stem cell for both lineages. This explains why similar signaling networks regulate the development and biological functions of these cells. One crucial regulatory mechanism involves interactions with soluble mediators of innate immunity, including activated elements of the complement cascade (ComC). For many years, ComC proteins were thought to be synthesized only in the liver and released into blood to be activated by one of the three proteolytic cascades. The regulatory effects of activated components of ComC on hematopoietic stem progenitor cells (HSPCs) and mature hematopoietic cells have been well demonstrated in the past. However, recent data indicate that complement proteins are also expressed in several cell types, including lymphocytes and innate immune cells. This intracellular complement network has been named the “complosome.” Recent evidence from our group shows that the complosome is also expressed in HSPCs and plays an important yet underappreciated role in the expansion, trafficking, and metabolism of these cells. We propose that the complosome, like its role in lymphocytes, is necessary for the optimal function of mitochondria in hematopoietic cells, including HSPCs. This opens a new area for investigation and potential pharmacological intervention into the complosome network in normal and malignant hematopoiesis.

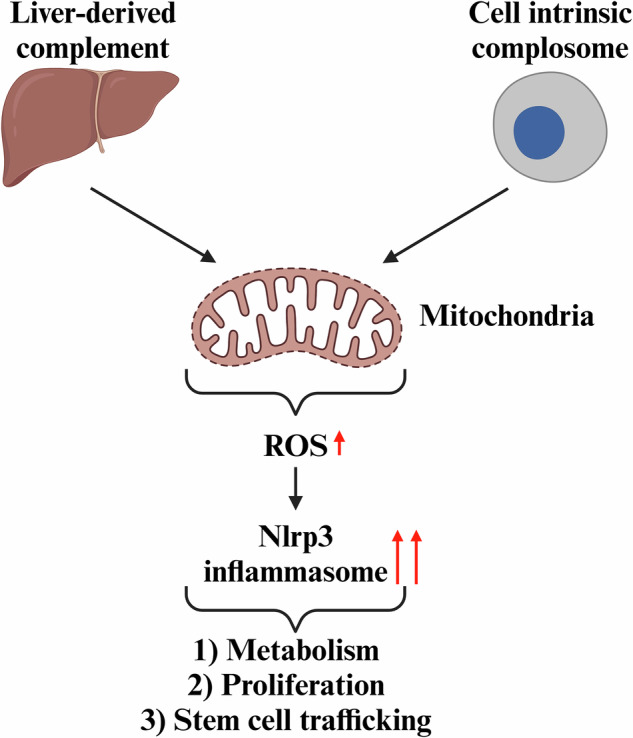

## Introduction

The innate immune system, also referred to as the nonspecific immune system, is an evolutionarily ancient primary defense mechanism found in plants, fungi, prokaryotic cells, and invertebrates. In vertebrates, it operates alongside the adaptive immune system, which consists of T and B lymphocytes, to fight infections and regulate various biological processes [1, 2]. In humans, it features a cellular component made up of leukocytes, macrophages, mast cells, eosinophils, basophils, dendritic cells, and γδ-T cells, as well as a soluble component comprised of around 50 different proteins and receptors from the complement cascade (ComC) [1]. The significance of the complement system is emphasized by the circulation of its proteins in plasma, alongside cell membrane receptors that account for roughly 10% of the serum gamma globulin fraction. Its primary function, as initially identified, is to enhance the efficacy of antibodies and phagocytes in removing invading microbes and damaged cells from the body. Components of the ComC that are synthesized in the liver are released as small interactive precursor proteins into circulation and are activated through three pathways: i) classical, ii) alternative, and iii) lectin pathways [1, 2].

In addition to its essential role in immune defense, innate immunity also regulates the development, trafficking, metabolism, and biological functions of hematopoietic cells directly at the level of i) hematopoietic stem/progenitor cells (HSPCs), ii) cells differentiating into major hematopoietic lineages, and iii) non-hematopoietic cells within the bone marrow (BM) microenvironment [1–3]. These significant effects driven by innate immunity influence both normal and malignant hematopoiesis.

Recently, our understanding of the innate immune system has expanded due to groundbreaking findings by Kemper et al. [4–6], which revealed that complement proteins are expressed in various types of cells. However, most research on the complosome has focused on lymphopoiesis. This intracellular complement network, called the “complosome,” regulates the biological responses and metabolism of lymphocytes. In CD4^+^ T cells, C3 storage has been identified in lysosomes, where it can be cleaved into C3a and C3b fragments by cathepsin L [4–7]. While intracellular C3a interacts with C3aR on lysosomes to activate the mTOR pathway and provide pro-survival signals for T lymphocytes, C3b interacts with CD46 (a complement regulatory receptor) on the cell surface to maintain T-cell homeostasis by preventing Notch-1-directed activation of these cells [4–7].

The complosome, initially discovered in CD4^+^ T cells, has now also been identified in CD8^+^ T lymphocytes, B cells, and cytotoxic CD8 + T lymphocytes [4]. Following this intriguing observation, research has shown that the complosome plays a role in regulating the responses of innate immune phagocytes [8]. Reports indicate that both human and mouse monocytes and macrophages rely on cell-autonomous and intracellular complosome activation to produce pro-inflammatory IL-1β during sterile inflammation. Identifying deficiencies in the complosome is crucial, as these deficiencies are linked to recurrent infections and contribute to atherosclerosis and arthritic diseases. Furthermore, it has been suggested that the complosome may play a role in various neurodegenerative and neuroinflammatory diseases, including multiple sclerosis, as well as in ulcerative colitis, and kidney pathologies [9–11].

These intracellular complement proteins are encoded by the same genes that produce liver-secreted complement. While most complosome proteins are generated directly within the cells, they can also be further internalized from extracellular sources and from the cell surface membrane [9]. For example, during infection, pathogens carry C3 covalently deposited onto them in the extracellular space inside the cell, activating innate immunity and mediating a restriction pathway that degrades the virus [12]. Studies in B-lymphocytes have revealed that C3 circulating in peripheral blood is able to enter the nucleus of viable B cells, suggesting its potential involvement in the regulation of gene transcription after interaction with histone proteins [13, 14]. Interestingly, no studies to date have reported the internalization of C5. This may be explained by the significantly lower concentration of this complement protein in peripheral blood compared to C3 [1, 2].

Despite this, certain complement elements can be internalized from blood, but this does not diminish the significant role of intracellularly expressed complement in regulating cell biology. Surprisingly, no research on the complosome has focused on normal and malignant hematopoiesis. Since hematopoietic cells and lymphocytes arise from stem cells that are shared by both lineages, we focused on the role of this intriguing intracellular network in hematopoiesis.

## Hematopoietic stem/progenitor cells, BM-stroma cells and leukemic blasts express complosome

Based on the expression data of the complosome in lymphocytes reported by Kemper et al. [4–6], we became interested in whether the complosome is also expressed in HSPCs [15]. To address this question, we isolated mRNA from purified human CD34^+^lin^-^CD45^+^ cells and observed mRNA expression for C3, C5, C3aR, C5aR1, and C5aR2 [15]. This data was subsequently confirmed by single-cell seqRNA. Similarly, we detected the expression of complement elements in purified murine Sca-1^+^lin^-^CD45^+^ bone marrow cells enriched for HSPCs [15]. Concurrently, we evaluated the expression of the complosome in murine bone marrow stroma cells under steady-state conditions, during pharmacological mobilization using granulocyte colony-stimulating factor (G-CSF) and the CXCR4 antagonist (AMD3100 - Plerixafor), and after myeloablative conditioning for hematopoietic transplant using γ-irradiation. We also found that the complosome is expressed in a “tonic” manner in bone marrow stroma cells, with its expression upregulated following the administration of pro-mobilizing agents and conditioning for transplantation via lethal irradiation [15].

Interestingly, we also detected the expression of the complosome in purified human stem cell populations, including BM-derived CD45^−^KDR^+^CD133+ endothelial progenitor cells (EPCs), CD45^−^KDR^+^CD133^−^CD73^+^ mesenchymal stromal cells (MSCs), and CD133^+^lin^−^CD45^−^ very small embryonic-like cells (VSELs) [16]. The expression of complosome mRNA elements, particularly C3 and C5, was significantly higher in the evaluated stem cells compared to BM mononuclear cells [15, 16]. Furthermore, complosome expression was notably upregulated in these cells after stimulation with stromal-derived factor-1 (SDF-1) [16].

We also investigated the expression of the complosome in several human leukemic cell lines and detected mRNA expression for C3, C5, C3aR, and C5aR1 in HEL, K-562, HL-60, HG-1a, and Nalm6 cells. The potential role of the complosome in regulating the biology of these cells was demonstrated after deleting the expression of C3 and C5 using the CRISPR-Cas9 strategy, which affected the migration of leukemic cells to SDF-1 and eATP (in preparation). Additionally, C3 and C5 mRNA from the complosome were highly expressed in AML blasts purified from peripheral blood. These data demonstrate that the complosome network is not only expressed but may also be involved in regulating both normal and malignant hematopoiesis.

## Complosome is activated in HSPCs and regulates their trafficking – differences between cells from C3-KO and C5-KO mice

Based on complosome expression data in HSPCs, we evaluated the biological effects of complosome deficiency using bone marrow cells purified from C3-KO, C5-KO, and C5aR1-KO mice as a model. Additionally, the complosome also includes C5aR2, which preferentially binds _desArg_C5a. The exact role of this C5a receptor remains somehow unclear [17, 18], and its function in the complosome network requires further study.

We observed that complement-deficient mice exhibit normal peripheral blood parameters under steady-state conditions; however, there was a significant decrease in the number of SKL cells and in the counts of clonogenic BFU-E, CFU-GM, and CFU-Meg progenitors in C5-KO mice [15]. In contrast, these parameters in C3-KO mice were similar to those seen in wild-type (WT) littermates. We also noted differences in the chemotactic responsiveness of C3-KO, C5-KO, and C5aR1-KO bone marrow (BM) cells to key chemotactic agents for hematopoietic stem and progenitor cells (HSPCs), including stromal-derived factor-1 (SDF-1), extracellular adenosine triphosphate (eATP), and sphingosine-1 phosphate (S1P) [19, 20]. While C3-KO BM cells show normal chemotactic responsiveness compared to WT cells, chemotaxis in C5-KO and C5aR1-KO BM cells was significantly reduced [15]. This provides evidence that the proximal part of complosome activation at the level of C3 differs from the distal part related to activation of C5.

Next, we conducted G-CSF- and AMD3100-induced mobilization in global complement and intracellular complosome-deficient animals. It turned out that C3-KO mice were effective mobilizers, releasing more cells, including HSPCs, into the blood [21]. This finding was further validated in C3aR-KO mice [21]. Furthermore, our studies with irradiated chimeras revealed that wild-type (WT) mice reconstituted with C3aR-KO derived bone marrow (BM) cells, unlike C3aR-KO mice reconstituted with WT BM cells, were more sensitive to G-CSF–induced mobilization [21]. These data confirm the significant role of C3aR expressed on HSPCs in our mobilization outcomes. In contrast to C3-KO deficient animals, the mobilization results in C5-KO mice differed [22]. These animals exhibited resistance to pharmacological mobilization by G-CSF and AMD3100, classifying them as poor mobilizers [22]. Similar results were noted in C5aR1-KO mice. Thus, we observed further differences among complosome-deficient animals, and we will address these findings in more detail later in this review.

In studying the trafficking of complosome-deficient HSPCs, we evaluated their homing and engraftment after transplantation into WT animals. Once again, we observed differences between BM cells purified from C3-KO and C5-KO mice. While C3-KO BM cells engrafted properly in WT mice [23], there was a significant reduction in homing and engraftment after the transplantation of C5-KO cells [24]. A similar defect was observed when C5aR1-KO cells were transplanted into WT littermates [15]. In these experiments, the number of donor-derived PKH67-labeled BMMNCs and the count of CFU-GM clonogenic progenitors, measured 24 hours post-transplantation in WT recipient mice, were decreased. Furthermore, day-12 colony-forming units in the spleen (CFU-S) and day-12 CFU-GM clonogenic progenitors isolated from BM were also present at lower levels after the transplantation of complosome-deficient cells compared to WT mice receiving WT BM cells. In parallel, we noted a slowdown in the recovery kinetics of leukocytes and blood platelets following the transplantation of C5-KO or C5aR-KO cells [15, 24]. These results indicate that the intracellular expression of C5 in HSPCs is essential for the in vivo migration, homing, and engraftment of transplanted HSPCs in the BM microenvironment.

## Oxygen consumption rate (OCR) studies in complosome-deficient murine bone marrow cells reveal differences in adaptation to oxidative stress between C3-KO and C5-KO cells

The role of intracellular C5a in human lymphocytes is linked to the regulation of the electron transport chain in mitochondria [7]. These intracellular organelles, as shown, express C5aR1 and respond to C5a stimulation. Additionally, mitochondria express C5aR2 and C3aR alongside C5aR1.

**Fig. 1:**
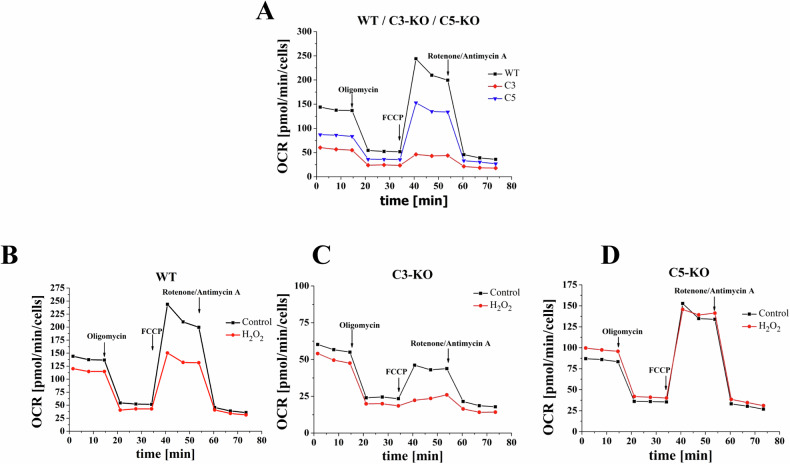
Mitochondrial oxygen consumption rate (OCR). Mitochondrial activity of lin^−^ cells isolated from the bone marrow of WT, C3-KO, and C5-KO mice exposed to 10 µM H_2_O_2_. **A** Compares OCR in cells isolated from WT, C3-KO, and C5-KO mice [25]. **B–D** Compare OCR in untreated cells and those treated with 10 µM H_2_O_2_ (**B** – WT, **C** – C3-KO, and **D** – C5-KO). Values are expressed as the mean ± standard deviation (SD) from at least three independent experiments (*n* = 3). Statistical analysis was performed using an unpaired method. This data is adopted from our already published original licensed under a Creative Commons Attribution 4.0 International License [25].

In our studies addressing the observed differences in the trafficking of murine complosome-deficient HSPCs, we conducted a metabolic analysis using plate-based live cell assays with a Seahorse extracellular flux analyzer [25, 26]. First, we evaluated the oxygen consumption rate (OCR) of lineage- BM cells (lin- BM) isolated from WT, C3-KO, and C5-KO during steady-state conditions and after exposure to mitochondrial function modifiers. Accordingly, we measured OCR: i) under steady-state conditions; ii) after exposure to the mitochondrial ATP synthase (complex V) inhibitor oligomycin; iii) followed by maximal stimulation of the respiratory rate induced by the uncoupler carbonyl cyanide-4-(trifluoromethoxy) phenyl-hydrazone (FCCP); and iv) finally, after exposing cells to Rotenone/Antimycin A, which inhibits mitochondrial respiratory chain complexes I and III, thus completely blocking the electron transport chain (Fig. 1) - adapted from our already published original licensed under a Creative Commons Attribution 4.0 International License [25].

The major findings from these oxygen consumption rate studies revealed that lin^−^BM cells isolated from complosome-deficient C3-KO and C5-KO mice under steady-state conditions exhibited significantly lower basal and maximal respiratory levels Fig. 1 [25]. This suggested compromised mitochondrial function. Additionally, C3-KO mice showed a complete inability to adapt to oxidative stress, as evidenced by a negative Spare Respiratory Capacity (SRC < 0). In contrast, C5-KO mice retained some ability to manage oxidative stress, indicated by a positive SRC (SRC > 0) [25]. These findings underscore the distinct differences in mitochondrial function between these two complement-deficient cell types, which may explain the variations in the trafficking of HSPCs mentioned earlier in this review.

Further experiments were conducted by exposing complosome-deficient cells to mild oxidative stress after treatment with 10 µM H_2_O_2_. Notably, H_2_O_2_ could be generated within stressed cells as one of the reactive oxygen species (ROS) produced in response to NADPH-oxidase 2 (Nox-2) activation [25, 27]. We observed that the SRC parameter, which measures adaptation to stress, increased in C5-KO mice during oxidative stress, suggesting an enhanced ability to adapt to environmental changes, as seen, for example, during sterile inflammation. Importantly, BM lin^−^ cells isolated from WT mice showed a significant decrease in SRC, indicating an even lower adaptive capacity than C5-KO animals [25]. Furthermore, we noted a decrease in SRC in C3-KO cells following H_2_O_2_ treatment, confirming a complete lack of adaptive response to oxidative stress, consistent with the results under steady-state conditions.

In conclusion, our OCR studies conducted under steady-state conditions and after exposure to mild oxidative stress revealed that lin^−^ BM cells isolated from C3-KO mice exhibit mitochondrial defects, as indicated by their impaired ability to adapt to oxidative stress. In contrast, lin^−^ BM cells derived from C5-KO mice show a high level of adaptation to this challenge [25]. Since stem cell trafficking is driven by sterile inflammation-mediated stress induced in BM and blood, this data explains why HSPCs from C3-KO mice are effective mobilizers and why their cells navigate more efficiently to the BM after transplantation compared to C5-KO counterparts, which adapt better to extracellular stress challenges. To explain these discrepancies, we must consider that in C3-KO HSPCs, complosome effects are mediated by C5, which remains intact in these mutant cells. Specifically, C5a generated after C5 cleavage is a potent activator of ROS release from mitochondria, effectively activating the Nlrp3 inflammasome [28, 29]. We have previously demonstrated that this intracellular pattern recognition receptor (PRR) is a major driver of HSPC trafficking [30, 31]. In contrast, in C5-KO cells, the main complosome network component is C3, which, when activated to C3a, promotes, as reported, the retention of HSPCs in BM niches. Consequently, these cells show impaired mobilization into blood. These cells also activate a less efficient Nlrp3 inflammasome [25] and, as a result, navigate poorly after transplantation to BM niches [24].

## Evidence that mitochondria function in HSPCs depends on complosome activity

Our oxygen consumption rate studies revealed mitochondrial defects in complement-deficient lin^-^BM cells. To further illuminate this data, we concentrated on specific mitochondrial functions in C3-KO, C5-KO, and C5aR1-KO cells [32]. Consequently, we measured lactate production and assessed the release of lactate dehydrogenase (LDH) from the cells, the enzyme that converts pyruvate to lactate [32]. This approach allowed us to explore the glycolytic pathway in these mutant cells more deeply. We also measured glucose uptake and examined mitochondrial physiology by using MitoTracker staining.

We observed that cells from complosome-deficient animals under steady-state conditions exhibited increased lactate production and enhanced lactate dehydrogenase (LDH) release [32]. These findings indicate the reliance of mutant HSPCs on anaerobic glycolysis. ATP production in C3-KO cells, in contrast to C5-KO and C5aR1-KO mice, was lower under steady-state conditions and did not significantly change after exposure to the mitochondrial-damaging agent hydrogen peroxide (H_2_O_2_). This suggests a greater dependence on anaerobic glycolysis in C3-KO cells compared to their C5-KO and C5aR1-KO counterparts [32]. Lastly, we assessed mitochondrial membrane integrity in the studied cells using MitoTracker green and deep red assays. Compared to WT cells, we found that mitochondria from complosome mutant lin-BM cells accumulated lower levels of the MitoTracker probes [32]. This assay indicates the presence of mitochondrial defects in the studied cells. We are currently conducting more detailed investigations to evaluate the expression and function of mitochondrial (I-V) complexes in complosome mutant animals.

## The Nlrp3 inflammasome is key to understanding the role of complement and the complosome network in HSPC trafficking and metabolism

The Nlrp3 inflammasome functions as an intracellular pattern-recognition receptor (PRR) and a sensor of host innate immunity, recognizing pathogen-associated molecular patterns (PAMPs) and danger-associated molecular patterns (DAMPs), including alarmins associated with sterile inflammation [33, 34]. Therefore, the Nlrp3 inflammasome is a crucial element of tissue homeostasis, facilitating the sterile, non-inflammatory removal of damaged cells and supporting tissue repair. Since sterile inflammation in the bone marrow is a key regulatory mechanism in pharmacological mobilization, as well as in the homing and engraftment of transplanted hematopoietic stem and progenitor cells (HSPCs) in myeloablated hosts, the activation of the Nlrp3 inflammasome plays a significant role in these processes [19]. The Nlrp3 inflammasome is activated by intracellular ROS, which is produced by Nox-2 activated mitochondria and cell membrane receptors [33, 34].

Two major regulators of sterile inflammation are i) activated complement cleavage fragments in PB, namely C3a, C5a, and C5b-C9 (membrane attack complex - MAC), and ii) ATP (eATP) released from stressed cells [1, 2, 19]. These signaling factors effectively increase the level of ROS in the cytosol once their corresponding receptors - C3aR, C5aR1, and P2X receptors - are activated. Furthermore, since the receptors for intracellular complement cleavage fragments (C3aR and C5aR1) and ATP receptors (P2X7 and P2X4) are located on mitochondria, their activation and the subsequent release of ROS also lead to the activation of the Nlrp3 inflammasome [35].

It has been shown that the activation of the Nlrp3 inflammasome plays a role in the trafficking and metabolic regulation of HSPCs [31, 34, 36]. However, we must consider that the biological effects of activating this PRR depend on the level of activation and are characterized by the phenomenon of hormesis. Hormesis refers to the biphasic response of cells to potentially harmful stimuli [37, 38]. At low doses, within the beneficial hormetic zone of activation, the effects of the Nlrp3 inflammasome are positive for cells, impacting aspects such as cell trafficking and metabolism. However, at higher activation levels, the Nlrp3 inflammasome can result in cell damage and cell death through pyroptosis [27–29]. Thus, all the previously mentioned positive effects of Nlrp3 inflammasome activation depend on the degree of its activation.

To explain the differing responsiveness of C3-KO and C5-KO complosome-deficient cells to pharmacological mobilization and in hematopoietic reconstitution assays, we noted that under steady-state conditions, Nlrp3 activation was lower in cells from both complosome mutant mice compared to WT; however, the lowest activation was observed in C5-KO cells. Next, we exposed complosome-deficient cells to C3a, C5a, and eATP, and studied Nlrp3-inflammasome activation using a caspase-1 activation-based glow assay. In WT cells, this exposure significantly increased activation [15, 25]. In C3-KO cells, eATP and C5a enhanced Nlrp3 inflammasome activation compared to C5-KO cells, consistent with the increased ROS production observed following treatment with these activators [15, 25]. Meanwhile, exposure to C3a had no effect. This aligns with our observations that C3-KO cells with intact intracellular C5 levels may generate C5a and thus are more sensitive to oxidative stress and exhibit enhanced Nlrp3-inflammasome-mediated HSPCs trafficking. Interestingly, it is worth mentioning that Nlrp3 inflammasome mutant mice have a statistically significant decrease in HSPCs in the BM, similar to those observed in C5-KO cells. This suggests that the C5a-Nlrp3 inflammasome axis is important in expanding the pool of these cells in the BM.

## Complosome-mediated effects on metabolism of HSPCs

The role of the complosome in metabolism has primarily been studied by Dr. Kemper’s group in lymphocytic cells [39]. As noted earlier, the complosome C3 cleavage fragment, C3a, activates the C3a receptor found on intracellular lysosomes, stimulating mTOR. The other cleavage fragment, C3b, is secreted from the cells and interacts with the complement regulatory protein CD46 receptor and the membrane cofactor protein on the cell surface [11]. While CD46 is an inhibitory complement receptor, its interaction with secreted intracellular C3b leads to: *i) activation* of certain metabolic enzymes, *ii)* increased influx of glucose and amino acids, and *iii)* activation of intracellular C5, which releases intracellular C5a to activate the Nlrp3 inflammasome, as discussed in the previous paragraph. Importantly, CD46’s contributions to the metabolic control of lymphocyte biology appear to be human-specific, as rodents do not express CD46 in somatic tissues [11, 40–42]. The murine protein that mimics the functions of human CD46 has not yet been discovered. Therefore, more studies are needed to understand the effects of the complosome on cell metabolism in murine HSPCs.

Based on these and other investigations, the role of the Nlrp3 inflammasome in regulating the complosome-mediated regulation of metabolism remains poorly understood. The primary mediators activated and released from the Nlrp3 inflammasome due to caspase-1 activation are interleukin-1 beta (IL-1β) and interleukin-18 (IL-18) [30]. Nlrp3-KO zebrafish cells have been reported to have impaired hematopoiesis [36]. Frame et al. identified a crucial role for the inflammasome in stimulating the de novo production of zebrafish and human hematopoietic stem and progenitor cells (HSPCs) [36]. IL-1β released from the Nlrp3 inflammasome activates hemogenic endothelium to promote the formation of embryonic HSPCs. Additionally, inflammasome activation enhances human HSPC expansion in human hemogenic cultures. In fact, some metabolic effects of IL-1β have been described previously, indicating that IL-1β modulates the activity of glycolysis in macrophages by increasing the potent driver of this pathway, 6-phosphofructo-2-kinase/fructose-2,6-bisphosphatase 3 (PFKFB3) [43].

To explain the metabolic effects of Nlrp3-inflammasome activation, we previously proposed a potential role for gasdermin pores in cell membranes that are formed in a caspase-1-dependent manner [44]. These pores facilitate the release of various DAMPs (alarmines) from cells, which interact with corresponding receptors on cell membranes, providing positive feedback to modulate metabolism. Recently, this notion has gained support. Studies have reported that gasdermin pores may encourage tissue repair and stimulate metabolism by releasing certain bioactive lipids (oxylipins) that enhance the regeneration of murine skeletal muscle cells in a fibroblast growth factor – fibroblast growth factor receptor-dependent manner. However, this requires that the gasdermin-mediated effects remain within a beneficial hormetic zone, as excessive and prolonged gasdermin formation may lead to detrimental outcomes, such as pyroptosis of these cells [33, 34]. Based on this data, further studies are necessary to investigate the effects of other alarmines released through gasdermin pores on the metabolism of HSPCs.

**Fig. 2:**
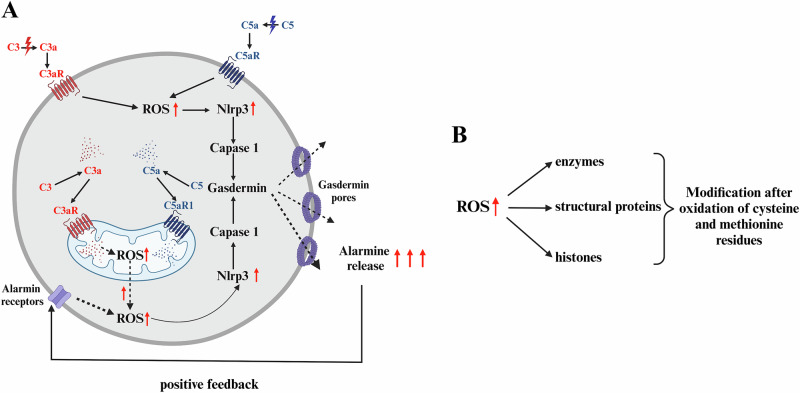
The effects of circulating and intracellular complement on HSPCs. **A** Active components of the complement cascade, such as C3a, C5a, and non-lytic C5b-C9 (MAC), interact with cell surface membranes that express receptors for C3a (C3aR) and C5a (C5aR1 and C5aR2). Additionally, complement proteins C3 and C5 are expressed intracellularly and are activated by enzymatic cleavage to generate C3a and C5a. The corresponding receptors, C3aR and C5aR1, along with C5aR2, are located on mitochondria. Activation of these receptors, whether on the cell surface [44, 51] or mitochondrial membranes [7, 8, 25] elevates the level of cytosolic ROS, a powerful activator of the Nlrp3 inflammasome. The Nlrp3 inflammasome, via caspase-1, processes IL-1β and IL-18 into their active forms, which are subsequently released from the cells. Simultaneously, gasdermin pores form on the cell membrane, releasing various alarmins from hematopoietic cells [44, 52]. If this release occurs within a beneficial “hormetic zone,” alarmins activate their specific receptors on the cell surface, affecting cell trafficking and metabolism while also contributing to an intracellular increase in ROS levels [44]. **B** The intracellular ROS, depending on their concentrations, may be either beneficial or detrimental to the cells [28, 29]. Within the beneficial hormetic range [37, 38], they modify various enzymes, structural proteins, and histones, influencing cell proliferation, metabolism, and trafficking [28, 29]. This figure was generated using BioRender.

We postulate, as summarized in Fig. 2, that complosome-mediated activation of the Nox2-ROS-Nlrp3 inflammasome axis may explain its effect on metabolism through caspase-1-induced gasdermin pore-mediated release of IL-1β, IL-18, and several alarmins (e.g., eATP, S1P, HMGB1) that regulate cell metabolism in response to stress.

To support the role of the complosome in the metabolism of HSPCs, we analyzed the expression of various enzymes involved in the metabolism of glucose, lipids, and amino acids in HSPCs from C5-KO and C5aR1-KO animals [15, 45]. We observed a decrease in the expression of enzymes associated with lipo- and steroidogenesis, including cholesterol, sterol regulatory element-binding protein 2 (SREBP2), 3-hydroxy-3-methyl-glutaryl-coenzyme A reductase (HMGCR), hydroxymethylglutaryl-CoA synthase (HMGCs), and acid sphingomyelinase (ASMAse). Stimulation of WT murine HSPCs, but not C5-KO HSPCs, activates the pentose phosphate pathway, which provides NADPH necessary for cholesterol and fatty acid synthesis, key components of cell membranes and membrane lipid rafts required for proper receptor signaling incorporated in this cell membrane, regulating HSPC trafficking and metabolism [45]. Moreover, membrane lipid rafts play a role in the release of extracellular microvesicles/exosomes from HSPCs that interact with surrounding cells in the hematopoietic microenvironment [46, 47].

Moreover, we noticed a reduced expression of essential enzymes involved in glycolysis: Glucokinase **(**GK), Glucose transporter 2 **(**GLUT2), Phosphofructokinase (PFKFB3), Glucose-6-phosphate dehydrogenase **(**G6PD), and large neutral amino acid transporter small subunit 1 (LAT1), which is a transmembrane amino acid transporter [15, 45]. The defect in GLUT2 expression in HSPCs isolated from C5-KO and C5aR-KO mice supports the impairment in glucose uptake in these cells. Additionally, the expression of these enzymes was also impacted after exposing C5 and C5aR1 deficient cells to the Nlrp3 inflammasome inhibitor MCC950 [15, 45]. This further highlights a connection between the complosome and the biological effects of Nlrp3 inflammasome activation.

## Conclusions and further research directions

Intracellular complosome networks have emerged as significant regulators of various cell types, including those from hematopoietic lineages. The discovery of the complosome in normal hematopoietic stem and progenitor cells (HSPCs) as well as in malignant leukemic cells opens new avenues for investigation [15]. However, several questions must be explored regarding whether complosome activation contributes to leukemogenesis or is merely a consequence of ongoing cell transformation. Other important inquiries related to hematopoiesis include the potential role of the complosome in initiating and sustaining graft-versus-host disease (GvHD) and inducing the cytokine storm seen during CAR-T cell therapy [48]. Additionally, we have limited knowledge about potential age-related and sex-dependent differences in complosome expression and activation. Furthermore, while the activation of circulating liver-derived complement in blood is well documented, intracellular activation of the complosome necessitates further research. A role for Cathepsin-L has been suggested for the cleavage of C3, and cathepsin D for C5 cleavage [49]. Moreover, in monocytes, the expression of mRNA encoding complement factors D (CFD) and B (CFB) has been identified, which together could assist in assembling complement alternative pathway convertases C3 (C3bBb) and C5 (C3bBbC3b) as potential candidates for intracellular complosome activation [8]. Both CFD and CFB are involved in activating an alternative pathway of ComC, with CFD cleaving and activating CFB. In contrast, factor H (CFH) inhibits the activation of C3 in macrophages, demonstrating that the complosome is regulated by “classic” ComC regulatory proteins [8]. We cannot discount the involvement of other intracellular proteases. Further studies are required to investigate the roles of C1q, a component of the classical pathway of ComC activation, and C5aR2 in the complosome network. Supporting this, C1q and C5aR2 receptors have been detected on mitochondria. More research is also needed to conduct precise metabolic studies in complosome-deficient cells and to explore the biological consequences of complosome activation in leukemic cells. It would also be crucial to examine the interaction of the complosome with other signaling pathways for which receptors are expressed on mitochondria [50]. In particular, the interaction between the complosome and receptors for purinergic signaling requires special attention, as the absence of mitochondrially expressed P2X7 produces a similar defect to C5aR1 deficiency [35].
